# In this issue, there are 10 articles: two review articles, six original articles, one clinical genomics, and one application note

**DOI:** 10.5808/GI.2019.17.1.e1

**Published:** 2019-03-31

**Authors:** Taesung Park

**Affiliations:** Department of Statistics, Seoul National University, Seoul 08826, Korea

The first review is about the procedures that are most commonly used for microbiome analysis and that are implemented in R packages. Recently, it has been well recognized that understanding the role of the microbiome and its modulation in human health is becoming increasingly relevant for preventive medicine and for the medical management of chronic diseases. The development of high-throughput sequencing technologies has made it possible to study microbial genomes and to perform a more precise quantification of microbiome abundances and function. In this review, Dr. Calle (University of Vic – Central University of Catalonia, Spain) provided a very excellent overview on the statistical methods of metagenomics data by placing particular emphasis on the compositional structure of microbiome data. Since microbiome data analysis involves high-dimensional structured multivariate sparse data due to its compositional nature, its analysis is very challenging. Dr. Calle presented the principles of compositional data analysis and distinguish between standard methods and those that fit into compositional data analysis.

The second review article by Ahn et al. (Sungkyunkwan University, Korea) overviewed diagnostic approaches and discusses current issues relating to neurocysticercosis (NC). NC is known to be caused by invasion of *Taenia solium* metacestode to the central nervous system. NC is an important neglected tropical disease and an emerging disease in industrialized countries due to immigration from endemic areas. Now, NC has a major global public health impact in terms of disability-adjusted life years. This review pointed out that modern imaging modalities have substantially improved the diagnostic accuracy of NC. Thus, NC is now controllable and manageable. However, the authors recommended further studies to investigate methods for controlling late-onset intractable seizures and for the serological diagnosis of NC patients infected with few worms.

This issue contains six Original Articles. First, Ham et al. (POSTECH, Korea) introduces a new transcriptome analysis pipeline for chronic obstructive pulmonary disease (COPD). The new pipeline uses a deconvolution process to reduce the heterogeneity of the sample. The new pipeline is shown to clearly identify the transcriptome data originated from the mild or moderate stage of COPD patients. Thus, this extensive evaluation of COPD transcriptome data could provide guidelines for analyzing heterogeneous gene expression profiles and classifying potential candidate genes that are responsible for the pathogenesis of COPD.

In the second article, Jo and Choi (Kangwon National University, Korea) investigated the notion of the functional relevance of the first intron sites by estimating amounts of rare alleles in the first intron. The proportions of rare single nucleotide polymorphisms of minor allele frequency in the first introns was estimated, and compared to those in other introns. The rare alleles were found to be significantly enriched in most of the regulatory marks locating in the first introns.

In the third article, Hwang et al. (Dankook University, Korea) presented an interesting study of athletic performance which is a complex multifactorial trait involving genetic and environmental factors. In particular, they analyzed mitochondrial DNA haplogroups to assess their association with the physical performance of Korean population. They successfully demonstrated that haplogroup F may play a crucial role in the physical performance of Korean athletes.

In the fourth article, Daoud (York University, Canada) proposes a window-based mechanism approach measure the seriousness of the difference among data-insights extracted from a composite biodata point. His approach is based on two components: undirected graph and Mosaab-metric space. He presents examples of influenza and Ebola viruses to demonstrate the robustness of his approach and to conduct comparisons.

The last two articles are about animal studies. Kim et al. (Korea Polar Research Institute, Korea) reported blood transcriptome resources of chinstrap (*Pygoscelis antarcticus*) and gentoo (*Pygoscelis papua*) penguins from the South Shetland Islands, Antarctica. The high-quality de novo assemblies of blood transcriptomes from these penguins were generated. Through Kyoto Encyclopedia of Genes and Genomes (KEGG) analyses they successfully detected many essential genes involved in the major innate immunity pathways. Blood transcriptome studies may be useful for checking the immune and health status of penguins without sacrifice.

Ramana (Dartmouth Medical School, USA) presented insights into the signal transduction pathways of mouse lung type II cells. Transcription factor profiling in microarray datasets revealed that several members of AP1, ATF, NF-κB, and C/EBP families involved in diverse responses were expressed in mouse lung type II cells.

In Clinical Genomics section, there is one article by Cho (National Cancer Center, Korea). He identified an ERBB pathway–activated cells in triple-negative breast cancer by using single-cell RNA sequencing data from a public resource. He showed that only the single-cell transcriptomes showed intratumor heterogeneity in the expression of three subtyping marker genes (ERBB2, ESR1, and PGR). Thus, his results show that ERBB signaling is activated via an indirect route and its molecular subtype is changed on a single-cell level.

In the Application Note section, one program HisCoM-mimi was introduced. HisCoM-mimi developed by Kim and Park (Seoul National University, Korea) was mainly for miRNA-mRNA integration analysis for binary phenotypes. HisCoM-mimi performs hierarchical structural component analysis to identify the miRNA-mRNA pairs associated with binary phenotypes. HisCoM-mimi uses the known mRNA target information provided by targetscan or mirTarbase into the integration model. Compared to the individual analysis of miRNA and mRNA, the integrated analysis by HisCoM-mimi is expected to higher detection power. Thus, HisCoM-mimi is expected to provide advanced accessibility to researchers who want to find the miRNA-mRNA pairs related with binary phenotypes.

